# Explaining Parenting Stress among Adoptive Parents: The Contribution of Mindfulness, Psychological Flexibility, and Self-Compassion

**DOI:** 10.3390/ijerph192114534

**Published:** 2022-11-05

**Authors:** Ana Luz Chorão, Maria Cristina Canavarro, Raquel Pires

**Affiliations:** 1 Faculty of Psychology and Education Sciences, University of Coimbra, Rua do Colégio Novo, 3000-115 Coimbra, Portugal; 2 Center for Research in Neuropsychology and Cognitive Behavioral Intervention, Faculty of Psychology and Education Sciences, University of Coimbra, Rua do Colégio Novo, 3000-115 Coimbra, Portugal

**Keywords:** adoption, adoptive parents, mindfulness, parenting stress, psychological flexibility, psychological resources, self-compassion

## Abstract

Although parenting stress has been identified as one of the most important and highly acceptable targets for postadoption psychological intervention, knowledge regarding the modifiable factors that contribute to explaining this outcome among adoptive parents remains scarce. This study aimed to explore whether and to what extent adoptive parents’ mindfulness, psychological flexibility, and self-compassion contribute to explaining parenting stress and to analyze whether this contribution varies according to children’s age, time passed since the adoptive placement, and the parents’ gender. Cross-sectional data from 302 Portuguese adoptive parents with children between 1 and 17 years old were collected online through self-response questionnaires. Controlling for a wide range of child-, adoption-, and parent-related variables, lower levels of mindfulness, psychological flexibility, and self-compassion were associated with higher levels of parenting stress. These associations were not moderated by children’s age, time passed since the adoptive placement, or the parents’ gender. The final regression model explained 50% of the variance in parenting stress. These results allow us to reflect on new guidelines for both preventive and remedial interventions with adoptive parents, given the apparent added value of promoting these psychological resources.

## 1. Introduction

Parenting stress has been defined as the stress resulting from an imbalance occurring when the perceived demands of the parents exceed their perceived resources [1]. When parenthood occurs under specific challenging circumstances, higher levels of parenting stress are expected [2]. This is often the case for adoptive parenthood. According to previous research, adoptive parents face specific developmental challenges that may leave them vulnerable to adverse outcomes [3]. One of these outcomes is parenting stress, which has been identified as one of the most important and highly acceptable targets for postadoption psychological intervention [4]. However, information regarding the modifiable factors that explain this outcome among adoptive parents remains scarce. To the best of our knowledge, none of the existing studies considered the potential contribution of parents’ psychological resources, such as mindfulness, psychological flexibility, and self-compassion, on parenting stress among adoptive families. Additional knowledge about this topic is needed to better inform adoptive family adjustment promotion through psychological intervention with adoptive parents.

### 1.1. Parenting Stress in Adoptive Families

Adoptive and biological parents experience similar parental tasks and individual challenges [3]. However, since the transition to parenthood, adoptive parents need to manage additional stressors, which may include coping with infertility, stigma about adoption, and uncertainty about the child’s arrival [3,5,6]. There is also an increased likelihood of their adoptive children having emotional and behavioral difficulties [6] due to their history of adversity [1]. As the children grow up, other adoption-related challenges may emerge, including introducing and discussing adoption with the child, helping them deal with adoption-related loss, and supporting and fulfilling the child’s curiosity about their origins [3]. Likewise, these parents seem to have less social support than nonadoptive parents [5]. These challenging parenting circumstances, denominated by [7] as “adoptive strains”, make adoptive parents more vulnerable than nonadoptive parents to experiencing stress in their parental role, which may also harm their children’s capacity to recover from previous adversity [8]. However, contradictory results were reported in previous research [9,10], suggesting a considerable variability of parenting stress among adoptive parents [8] that must be investigated to allow accurate intervention planning for those adoptive parents who are more likely to struggle either immediately or in the future [11].

According to parenting stress models (e.g., [11,12]), parenting stress can be explained by characteristics related to the external context (e.g., social support), to the child (e.g., age, gender, history of adversity), to the parent–child interaction (e.g., family dynamic), and, of particular relevance for the scope of this study, to the parents (e.g., adjustment to the parental role, emotional states, psychological resources [13]. However, research about the explicative factors of parenting stress among adoptive parents is limited, especially those exploring the role of parent-related variables [14]. In fact, although parent-related variables associated with parenting stress may be highly relevant in informing postadoption services, they have been receiving less attention than a child- or adoption-related variables [14]. The child’s age [15], gender [10], history of adversity [1], time spent in institutional care [9], age at adoptive placement [16], special needs [17], and behavioral problems [15,18,19] are the most studied child- and adoption-related variables as predictors of parenting stress. Only recently have a few studies assessed the explanatory role of adoptive parent-related variables in their own parenting stress (e.g., [11,14]). Parents’ age [20], educational level [21], socioeconomic status [22], and mental health status (e.g., depressive symptoms) [16] are the most studied. Despite the undeniable value of this body of research, the investigation into the role of adoptive parents’ psychological resources, such as mindfulness, psychological flexibility, and self-compassion, on parenting stress may be essential since these are individual resources that can be improved and modified through psychological intervention. According to a previous study using a Portuguese sample of adoptive parents, 93% of the participants would consider the provision of postadoption psychological intervention that includes the global promotion of parents’ well-being and specific components related to parenting stress useful. Approximately 76% of participants would be willing to participate in this type of intervention [23]. If the psychological resources under study prove to be explicative of parenting stress levels, they may constitute important targets for these kinds of interventions.

### 1.2. Parents’ Psychological Resources and Parenting Stress

According to Leeming and Hayes, when parents can apply psychological resources, such as mindfulness, psychological flexibility, and self-compassion, to their daily living, they tend to be psychologically healthier and more capable of promoting healthier family environments [24]. Mindfulness is a mental state defined by moment-to-moment, nonjudgmental awareness that lets individuals acknowledge and accept their emotions, thoughts, and bodily sensations [25]. Parents who can apply mindfulness to their parenting are more likely to be less reactive and more patient when dealing with challenging child behaviors. Furthermore, by opening parents’ awareness, mindfulness could help parents notice and genuinely appreciate children’s pro-social behaviors [26,27].

Psychological flexibility refers to an individual’s capacity to accept aversive emotional experiences while maintaining engagement in value-based behaviors [28]. A study conducted by Williams and colleagues found that parental psychological flexibility leads to lower levels of parenting stress and greater psychological flexibility in children, which, over time, can result in lower levels of psychopathology and higher levels of pro-social behavior in children [29]. Finally, self-compassion can be defined as “being open to and moved by one’s own suffering, experiencing feelings of caring and kindness toward oneself, taking an understanding, non-judgmental attitude toward one’s inadequacies and failures, and recognizing that one’s own experience is part of the common human experience” [30] (p. 87). When applied to parenting, self-compassion may reduce parenting stress, particularly by realistically adjusting expectations and goals regarding what parents expect of themselves as parents and of their children while accepting and comforting themselves for the loss of expectations once created. It can also allow parents to be compassionate with themselves and their children in challenging moments by understanding that these moments are part of the human experience and by decreasing rumination about parenting difficulties [31].

Although the literature about the relationship between these and other related psychological resources and parenting stress in adoptive families is scarce or nonexistent, research conducted with community samples [32,33] and parents in specific challenging conditions (e.g., children diagnosed with psychological or physical health conditions) [34,35,36] supports the relevance of exploring this theme among adoptive parents. According to these studies, self-compassion and psychological flexibility are negatively correlated with parenting stress under various challenging parenting circumstances usually present in adoptive parenthood [35,37]. Several reviews and meta-analyses also found that mindfulness-based interventions could effectively manage parenting stress under diverse, challenging parenting circumstances [24,34,36,38]. Such findings are not surprising since, as noted by Leeming and Hayes, mindfulness, self-compassion, and psychological flexibility, as a coherent set of crucial skills, have the power to produce positive changes in the families’ systems [24].

Although we are not aware of any related study in the adoption field, in the specific case of adoptive parents, psychological resources such as mindfulness, psychological flexibility, and self-compassion are expected to be particularly beneficial if considering the abovementioned increased challenges that these parents are faced with and the harmful impact that parenting stress can have on adopted children [39]. It is well known that children who encounter traumatic experiences such as emotional and/or physical abuse have difficulties creating a secure attachment with their caregivers [39]; they can experience other severe psychological and emotion-regulation consequences associated with previous adversity [40], and these challenging circumstances may predict parenting stress [39,41]. 

However, the relationship between children’s insecure attachment patterns and parenting stress seems to be a reciprocal process since high levels of parenting stress also seem to lead to an increase in insecure attachment patterns or a decrease in secure attachment patterns in children [8]. Mindfulness could help adoptive parents be less reactive to the child’s challenging behaviors related to these circumstances and truly enjoy the moments of family harmony. Psychological flexibility may also be helpful, allowing parents to stay focused and behave in a way that is in line with their values and goals even though they are facing aversive emotional states. Additionally, self-compassion may enable parents to accept these challenging moments and the emotions that emerge from them as part of the human experience, especially given the increased challenges that emerge from this type of parenting. Self-compassion may also help adoptive parents cope with the usual discrepancy generated between their idealization of parenthood and of their child and the real experience.

### 1.3. The Role of Children’s Age, Time Passed since the Adoptive Placement, and Parents’ Gender

In addition to the lack of literature on the impact of parents’ psychological resources on parenting stress among adoptive families, important gaps arise in the nonspecific literature that prevents us from generalizing its findings to the adoption field without further investigation.

The first concerning gap in the literature is that most of the studies regarding parenting stress have only considered parents of children of specific age groups (e.g., adolescence) [40,42]. As different children’s ages pose different parenting challenges and can imply specific adjustment mechanisms for parents [43], the choice to study samples including only parents of children of specific age groups disables investigation of the role played by parents’ psychological resources in dealing with parenting stress at different developmental stages of the child. It is also important to note that among adoptive families, most parenting stress studies have focused on specific phases of the family life cycle (e.g., the first years after adoption) [11,16]. However, in addition to the challenges usually associated with the different stages of a child’s growth and the life cycle of an adoptive family, there are several emerging parenting challenges that may be confusing, stressful, and exhausting [8,43], requiring different psychological resources from parents to maintain their well-being in different phases of the family life cycle. Finally, parenting stress research has also been primarily focused on mothers, with fewer studies (e.g., [11,44,45]) fully considering the role of fathers. In fact, there are various individual, biological, and cultural gender differences that can influence how fathers and mothers address the challenges that emerge from parenthood [46]. Including adoptive fathers in the study of these phenomena may allow us to investigate how the parents’ gender influences the relationship between their psychological resources and the levels of parenting stress.

### 1.4. The Present Study

Taking into account the vulnerability of adoptive parents to experience parenting stress [3], how parenting stress may impact the healthy development of their children [12,39,41], and the importance of identifying parents’ modifiable psychological resources that may contribute to preventing or reducing parenting stress to target them in psychological postadoption interventions, the main aim of this study is to explore whether, and to what extent adoptive parents’ mindfulness, psychological flexibility, and self-compassion contribute to explaining parenting stress. We hypothesize that even after controlling for the child-, adoption-, and other parent-related variables, adoptive parents with higher levels of mindfulness, psychological flexibility, and self-compassion would experience lower levels of parenting stress. Additionally, we intend to explore whether the contribution of these psychological resources to explaining parenting stress varies according to children’s age, the time passed since the adoptive placement, and the parents’ gender. Given the lack of literature on this topic, we did not elaborate on specific hypotheses.

Beyond enabling the identification of adoptive parents with a higher probability to struggle either immediately or in the future, our study could also contribute to a better and more effective psychological intervention design by identifying specific therapeutic targets to address that might prevent or reduce parenting stress according to each of the family statuses regarding child’s age, time passed since the adoptive placement, and parents’ gender. As already noted, this type of intervention, if effective, can affect not only parents’ well-being but also their children’s full development and health, the ultimate goal of child adoption.

## 2. Materials and Methods

### 2.1. Procedures

This cross-sectional study is part of a larger research project entitled “A Mindfulness approach to adoptive parents’ psychological and parenting functioning: Comprehensive analysis and evaluation of a post-adoption psychological intervention”. This project was approved by the Ethics Committee of Faculty of Psychology and Education Sciences of the University of Coimbra. Data collection occurred between September 2020 and June 2021. An online self-report assessment protocol developed for the project hosting this study was used through the LimeSurvey platform (a secure online tool provided by the host institution). The eligibility criterion for inclusion in the study was having at least one adopted child under 18 years old. Parents who had more than one adopted child under 18 years old were instructed to complete the assessment on the child with whom they experienced more difficulties. Participants were recruited through all Portuguese governmental adoption agencies. The agencies sent an email to potential participants inviting them to participate in the study. This email contained summarized information about the study’s objectives, the researchers’ contacts, and the informed consent and online questionnaire link. Participation in the study was voluntary and unpaid. Confidentiality was ensured for potential participants.

As dependent observations (i.e., parental dyads) were not sufficient in number for dyadic analysis, only independent observations (i.e., one parent per family) were used in the present study. The independence of the observations among dual-parent adoptive families was ensured by alphanumerical codes provided by the participants and containing first and last name initials, birth dates (day and month), and email indexes of both members of the parental dyad. Data from participants with no dyad correspondence in the database were coded as independent observations and were the only data used in the present study. After sample selection, participants’ data were irreversibly anonymized. Electronic consent was obtained from all participants by selecting the option “Yes, I authorize” after reading the information about the research project, the inclusion criteria, the researcher’s duties, the participant’s rights, and the data protection policy used for data storage.

### 2.2. Measures

Sociodemographic, health, and adoption-related data were assessed through a questionnaire developed by the authors based on a comprehensive literature review and revised by key stakeholders in the adoption and parenthood fields.

#### 2.2.1. Sociodemographic, Health, and Adoption-Related Data

Sociodemographic information included parents’ age, sex, educational level, professional status, marital status, number of household members, household income, family type (only adopted children vs. both adoptive and biological children), and child’s age and sex. Health-related information included parents’ history of mental health (e.g., history of mental illness) and physical health (e.g., history of physical health problems) and their child’s history of health problems (e.g., physical or mental disability; diagnosed psychological/psychiatric problems). Adoption process information included the application type (couple vs. single), adoption type (domestic vs. international), number of children adopted at the same time (e.g., one child, two siblings), child’s age at adoptive placement, years since the adoptive placement occurred, number of years the child had been in foster care, reason for foster care measure, and foster care situation before the child was adopted. Since the sample collection took place during the COVID-19 pandemic, we considered the literature’s reported impact of the pandemic on parents’ emotional adjustment (e.g., [47]), which could interfere with the phenomena under study, and therefore, parents were also asked about COVID-19-related aspects (e.g., whether they were or had been infected; whether they had been identified as an at-risk population (i.e., being older than 60 years and/or having health conditions like lung or heart disease, diabetes or conditions that affect the immune system [48]); the perceived impact of COVID-19 in their lives and in their child’s lives).

#### 2.2.2. Parenting Stress

The Portuguese version of the Parenting Stress Index—Short Form (PSI-SF [49,50]) was used to evaluate parents’ parenting stress. The PSI-SF is a self-response instrument that assesses parents’ general stress levels when performing their parental role. It consists of 36 items organized into three subscales: parenting difficulties (to assess the extent to which the parent is experiencing stress in their parenting role; e.g., “To be able to respond to my children’s needs, I end up depriving myself of having my own life”), dysfunctional parent–child interactions (to assess the extent to which the parent feels that their child does not meet their expectations and that interactions with them are not satisfactory; e.g., “When I take care of things for him or her, I get the feeling that my effort is not much appreciated”), and child difficulties (to assess how easy or difficult the parent perceives the child to be; e.g., “My child makes more demands on me than most children”). To adapt the questionnaire for the adoptive parent population, Items 4 and 5 were presented, respectively, as “Since this child was born/Since I adopted this child, I have never been able to do new and different things” and “Since this child was born/Since I adopted this child, I feel I cannot do the things I like”. Items were answered on a five-point Likert scale (1 = disagree entirely to 5 = agree entirely). A higher total score indicated higher parenting stress levels. The Portuguese version has adequate internal consistency levels, ranging from 0.71 (child domain) to 0.82 (parent domain). The total score has a Cronbach’s alpha of 0.89, illustrating good psychometric qualities. In this study, only the total score was used. Cronbach’s alpha in the present study was 0.95.

#### 2.2.3. Parents’ Mindfulness

The Portuguese version of the Mindful Attention and Awareness Scale (MASS [51,52]) was used to assess parents’ mindfulness. The MAAS is a self-response measure of mindfulness at the trait level. It consists of 15 items (e.g., “I find it difficult to stay focused on what is happening in the present moment”; “I seem to function on autopilot, without much conscious attention to what I am doing”) answered on a six-point Likert scale (1 = almost always to 6 = almost never). According to the original authors [50], the items on the scale determine the presence or absence of attention to and awareness about what is happening in the present moment. The scale’s total score is obtained by summing the fifteen items, with higher scores indicating a higher level of mindfulness.

The Portuguese version has good psychometric properties with a Cronbach’s alpha coefficient of 0.90, making this instrument reliable for measuring mindfulness traits. The Cronbach’s alpha in the present study was 0.94.

#### 2.2.4. Parents’ Psychological Flexibility

Parents’ psychological flexibility was assessed through the Portuguese version of the Acceptance and Action Questionnaire-II (AAQII [53,54]). The AAQII is a seven-item self-report measure to assess psychological inflexibility (e.g., “My painful experiences and memories make it difficult for me to live a life that I value”; “My worries get in the way of my success”). Items were answered on a seven-point Likert scale (1 = never true to 7 = always true). The scale’s total score is calculated by summing the seven items. Higher scores indicate lower levels of psychological flexibility. The Portuguese version has a good level of internal consistency, with all the samples in the instrument’s validation study showing Cronbach’s alpha coefficients over 0.89. The Cronbach’s alpha in the present study was 0.93.

#### 2.2.5. Parents’ Self-Compassion

The Portuguese version of the Self-Compassion Scale-Short Form (SCS-SF [55,56]) was used to assess parents’ self-compassion. The SCS-SF is a 12-item self-report measure of self-compassion organized into six subscales: self-kindness (e.g., “When I feel down, I tend to fixate and obsess about everything that is wrong”), self-judgment (e.g., “I am intolerant and not very patient about aspects of my personality that I do not like”), common humanity (e.g., “When I feel inadequate in some way, I try to remember that most people sometimes feel the same way”), isolation (e.g., “When something upsets or saddens me, I try to maintain my emotional balance [control my emotions]”), mindfulness (e.g., “When I fail at something that is important to me, I martyr myself with feelings of inadequacy”), and overidentified (e.g., “I disapprove myself and make judgments about my mistakes and inadequacies”). This instrument uses a five-point Likert scale (1 = almost never to 5 = almost always). It is structured to allow for the calculation of the scores for each subscale and the total self-compassion score, where higher scores indicate a higher level of self-compassion. Based on the instructions from the authors of the instrument, some items were reverse-scored. The Portuguese version has good psychometric properties, with good internal consistency (Cronbach’s alpha coefficients between 0.75 and 0.90), test-retest reliability (r = 0.78), and convergent validity (assessed by comparing the scale’s results to the results of the Portuguese versions of the General Health Questionnaire [GHQ-28], the Social Comparison Scale [SCS] and the Other as Shamer Scale [OAS]), making it a reliable instrument for assessing self-compassion. Only the total score was used in this study. The Cronbach’s alpha in the present study was 0.87.

### 2.3. Data Analysis

The data were analyzed using the Statistical Package for Social Sciences, v25 (IBM Corp., Armonk, New York, NY, USA). Descriptive statistics were first provided for all sociodemographic, health, and adoption-related covariables, study variables (i.e., parents’ mindfulness, psychological flexibility, and self-compassion), and the study outcome (i.e., parenting stress). To measure the association between sociodemographic, health, and adoption-related covariables and study variables, Kendall’s coefficient of rank correlation tau-sub-b, point-biserial correlation coefficient, and Pearson correlations (small effects: r ≥ 0.10; medium effects: r ≥ 0.30; large effects r ≥ 0.50) [57], were computed. Except for the hypothesized moderators of the association between study variables and parenting stress (i.e., children’s age, time passed since adoptive placement, and parents’ gender), only the sociodemographic, health, and adoption-related variables that were significantly associated (*p* < 0.05) with parenting stress were included as covariables in the following regression analyses. 

Considering the sample size and the number of potential explicative variables, two hierarchical regression models were then built to assess the independent contribution of each study variable to the study outcome (i.e., parenting stress) while controlling for sociodemographic, health, and adoption-related covariables, termed the main effects model: the first corresponding to the exploratory model where all the independent variables significantly associated with the outcome were entered into four blocks according to their conceptual nature; the second corresponding to the final model, where only the significant explicative factors of the exploratory model were entered according to the same blocks; significant effects corresponding to *p* < 0.10 were reported. To analyze the possible moderating effect of children’s age, time passed since adoptive placement, and parents’ gender on the association between each study variable and parenting stress, the computational tool PROCESS, version 3.5.2, model 1 [58], was used. A simple moderation model was run for each of the potential pairs of moderator and psychological resources under study (entered as independent variables), always entering parenting stress as the dependent variable. Thus, nine simple moderation models were created. In each model, sociodemographic and clinical characteristics of parents and children and adoption-related characteristics were entered as covariables if they were found to be significantly associated with parenting stress in the main effects model (*p* < 0.10).

## 3. Results

### 3.1. Sample

The sample of the present study was a partial sample of the larger research project and consisted of 302 Portuguese adoptive parents (independent observations) with a mean age of 47 years. Detailed information about the sociodemographic and health characteristics of the parents and their children, as well as data related to the adoption process, are presented in Table 1.

### 3.2. Associations with Parenting Stress

The descriptive statistics and Pearson’s correlations for the variables under study are presented in Table 2. Medium to large associations were found among all the variables under study. Parenting stress was negatively associated with mindfulness, psychological flexibility, and self-compassion. Mindfulness was positively associated with self-compassion and psychological flexibility. Self-compassion was positively associated with psychological flexibility.

### 3.3. Explicative Factors for Parenting Stress

Regarding sociodemographic, health, and adoption-related information, parenting stress was positively associated with parents’ educational level (r = 0.14, *p* = 0.037), diagnosis of mental health problems (r = 0.17, *p* = 0.004), and diagnosis of physical health problems (r = 0.11, *p* = 0.004), child’s age (r = 0.17, *p* = 0.003), severe behavior problems (r = 0.37, *p* < 0.001), diagnosed psychological/psychiatric problems (r = 0.32, *p* < 0.001), and age at the adoptive placement (r = 0.13, *p* = 0.031). In contrast, parenting stress revealed a negative association with the perceived impact of COVID-19 on both the parents’ (r = −0.25, *p* < 0.001) and the child’s lives (r = −0.32, *p* < 0.001). All significant associations with parenting stress showed medium to large effects, except for parents’ educational level, diagnosis of mental health problems, diagnosis of physical health problems and the child’s current age and age at adoptive placement, which showed small effects.

As shown in Table 3, when considered with no other variables in the model, parents’ educational level, diagnosis of mental health problems, and perceived impact of COVID-19 on their own life (step 1) significantly explained 8% of the variance in the outcome (F_(4, 291)_ = 7.78; *p* < 0.001). When the child’s severe behavior problems, diagnosis of psychological/psychiatric problems, and perceived impact of COVID-19 on the child’s life were added to the model (step 2), the perceived impact of COVID-19 on the parents’ life became non-significant, and the variables in the model explained 27% of the variance in the outcome (F_(7, 288)_ = 16.17, *p* < 0.001). Adding parents’ mindfulness, psychological flexibility, and self-compassion to the model (step 3), parents’ educational level and diagnosis of mental health problems became non-significant, and the model explained 47% of the variance in the outcome (F_(10, 285)_ = 27.35, *p* < 0.001). Finally, with the addition of the parents’ gender, the child’s age, and the time since the child’s adoptive placement (step 4), all the variables in the model accounted for 48% of the variance in the outcome (F_(13, 282)_ = 22.04, *p* < 0.001).

The final model, including only the significant contributors (*p* < 0.10), was significant (F_(7, 294)_ = 41.43, *p* < 0.001) and explained 49% of the variance in parenting stress. The child’s older age, presence of severe behavior problems, presence of a diagnosed psychological/psychiatric problem, and a more negative impact of COVID-19 in the child’s life, as well as lower levels of parents’ mindfulness, psychological flexibility, and self-compassion, significantly explained higher levels of parenting stress. All the hypothesized interactions tested through moderation models did not prove to be significant (data not shown).

## 4. Discussion

The present study aimed to explore whether and to what extent adoptive parents’ mindfulness, psychological flexibility, and self-compassion contribute to explaining parenting stress and to analyze whether this contribution varies according to the child’s age, time passed since adoptive placement, and parents’ gender. The results support our hypothesis by demonstrating that higher levels of parents’ mindfulness, psychological flexibility, and self-compassion were associated with lower levels of parenting stress; this contribution did not vary according to the child’s age, the time passed since adoptive placement, or the parents’ gender. These results are innovative and contribute to a better understanding of the relationship between adoptive parents’ psychological resources and parenting stress; they add pertinent new information for planning future research and a more effective clinical practice with this population, as will be discussed below.

### 4.1. Parents’ Psychological Resources and Parenting Stress

Our results showed that higher levels of parents’ mindfulness, psychological flexibility, and self-compassion were associated with lower parenting stress levels among our sample’s adoptive parents. These results corroborate previous studies conducted with parents in other challenging circumstances [34,35,36], adding to this body of literature evidence about the relevance of these variables in the specific population of adoptive parents. Individuals who experience higher levels of parenting stress tend to react automatically, impulsively, and negatively when interacting with their child, which activates the child’s defense system [59], leading to increased conflict and contributing to even higher levels of parenting stress. Indeed, mindfulness, psychological flexibility, and self-compassion seem to be resources that may promote an approach contrary to this one, which may prevent or break dysfunctional cycles of child–parent interaction. According to previous literature, when applied to the exercise of parenthood, these psychological resources may reduce the levels of parental emotional reactivity, increase parental practices in line with parents’ values, and promote the awareness of suffering as part of the human experience [26,31]. Thus, these skills may promote family harmony, leading to a lower frequency of stressful situations for parents; on the other hand, the fact that parents have these skills exercised may lead to better management of challenging situations within the family, thus contributing to lower levels of parenting stress.

### 4.2. Child-, Adoption-, and Other Parent-Related Variables and Parenting Stress

It is particularly important to note that the explicative role of adoptive parents’ psychological resources on their parenting stress occurred while controlling for a wide range of child-, adoption-, and other parent-related variables. These data add to previous adoption-specific literature in which, despite the expected variability of parenting stress levels as a function of these covariables, the explanatory power of the psychological resources under study seems to extend to a wide range of conditions. The hierarchical nature of the regression revealed that some of these child-, adoption-, and other parent-related variables also contribute to explain parenting stress. In contrast, others lost their explicative role after including parents’ psychological resources in the model.

The results of our study have shown that, in addition to the parents’ psychological resources, the child’s age, severe behavioral problems, and diagnosis of a psychological or psychiatric problem were positively associated with parenting stress. Conversely, the parents’ perceived impact of COVID-19 on the child’s life was negatively associated with parenting stress. In other words, older age, severe behavioral problems, diagnosed psychological or psychiatric problems of the child and a more negative perceived impact of COVID-19 in the child’s life were associated with higher levels of parenting stress in our sample. Concerning the child’s age, the results obtained in this study are consistent with those obtained by Farr and colleagues, who found that adoptive parents of older children reported higher levels of parenting stress [60]. A possible interpretation of these results is that as they grow up, in addition to the challenges inherent to greater freedom, autonomy, and independence, adopted children may also become more aware of their adoption context and feel a greater need to know more about their family of origin. This can lead to the emergence of family conflicts and a sense of insecurity in adoptive parents, increasing parenting stress levels [43].

Regarding the presence of the child’s severe behavioral problems, the results of this study are also consistent with previous literature [9,11,19,60,61]. In fact, children’s behavior problems have been recognized as the most important predictor of parenting stress [14]. The relationship between child behavior and parenting stress is a reciprocal process [62], where the existence of more disruptive behaviors in children leads to increased stress levels in the parents, who in turn will react impulsively, which further increases the child’s disruptive behaviors. Similarly, the literature reports the perception of the existence of emotional difficulties in the child as one of the predictors of parenting stress [16,60]; in this study, the results also showed that the existence of a child’s diagnosis of a psychological or psychiatric problem relates to higher levels of parenting stress.

Given the temporal context in which the sample for this study was collected, it became necessary to control for the effect of parents’ perceived impact of COVID-19 on their children’s lives, given the possible interference of this public health context in several variables under study, particularly the outcome. In fact, the association between the negative impact of COVID-19 on children’s lives (as perceived by their parents) and higher levels of parenting stress found in our study corroborate previous research on the impact of COVID-19 on parenting stress levels [63,64,65]. This association can be justified by the fact that the pandemic led to changes in children’s daily structure and routines, worries about COVID-19, and homeschooling [63]. These factors appear to have contributed to increased stress levels in children associated with the emergence of more disruptive behaviors, which in turn led to increases in levels of parenting stress. However, our results showed that even after controlling for the perceived impact of COVID-19 on children’s lives, the psychological resources under study contributed to explaining the levels of parenting stress. This leads to the conclusion that the explicative role of these psychological resources seems to be pertinent independently of other extraordinarily challenging circumstances in the family’s life.

Additionally, parents’ educational level and mental health problems, which were positively associated with parenting stress, became nonsignificant contributors to parenting stress after considering the role of parents’ psychological resources. This result is in line with previous findings, indicating that a higher educational level [21] and the presence of mental health problems in parents [16] may contribute to higher levels of parenting stress. However, this result also adds to previous research that lower levels of mindfulness, psychological flexibility, and self-compassion may be better explicative factors of higher levels of parenting stress than these variables. These results are particularly relevant since these psychological resources are modifiable variables in a therapeutic context, in contrast to parents’ educational level. In addition, these results also point to the possibility of using these psychological resources as specific therapeutic targets for preventing and treating high levels of parenting stress among individuals diagnosed with mental health problems. However, future studies must investigate these hypotheses to analyze them properly.

### 4.3. The Role of the Child’s Age, Time Passed since Adoptive Placement, and Parents’ Gender

Finally, the nonsignificant results regarding the potential moderating role of the child’s age, time passed since adoptive placement, and parents’ gender on the association between parents’ mindfulness, psychological flexibility, and self-compassion and parenting stress suggest that the explanatory role of parents’ psychological resources may extend itself to a wide range of conditions. However, they should be explored in samples of other natures and sizes in future investigations (e.g., samples including a greater proportion of male parents, dyadic samples, or larger samples of independent observations). Nevertheless, these results highlight the potential usefulness and cross-cutting nature of promoting these three psychological resources with adoptive parents, as they appear to help reduce stress in different personal and family contexts in both women and men, regardless of the age of their adopted children and regardless of how many years ago the adoptive placement occurred.

### 4.4. Limitations and Strengths

Despite this study’s innovative and important findings, some limitations must be considered.

First, it is relevant to note that its cross-sectional design does not allow us to establish the direction or causality of the associations between the variables. For example, high levels of parenting stress may be associated with a lower ability to be mindful and to experience favorable levels of psychological flexibility and self-compassion. Alternatively, these psychological resources may also act as protective factors for parenting stress, not allowing parents to experience such high levels of parenting stress in challenging interactions with their children. Likewise, the child’s emotional and behavioral difficulties might be a consequence of the stress experienced by their parents and a source of more parenting stress [11]. Thus, although it is agreed that cross-sectional exploratory studies are the first line of study for unexplored phenomena in a given population, the results of our study must be interpreted with caution, and future longitudinal studies are needed to clarify the direction of the associations between these variables and explore the explanatory mechanisms.

Second, it is important to mention several study characteristics that may compromise the representativeness of our results, as they may have led to a self-selected sample. Specifically, our sample was collected online (by filling out an online questionnaire). Parents with high levels of comprehension and more regular internet access may have filled out the questionnaires more frequently than their peers. We tried to overcome this limitation by (1) using a simple language for questionnaire dissemination, (2) providing different types of contacts for clarification of doubts and technical support in accessing the online protocol (e.g., email, phone, mail address), and (3) requiring simple procedures to access the questionnaires (e.g., one click only, universal browser access to the questionnaires without needing to install any additional software or functionality). Additionally, due to the aims of the research project in which this study is included and the clear way the aims were explained to potential participants, adoptive parents who had more interest, experiences, or needs in the respective topics probably participated more frequently than those who did not. We tried to overcome this limitation by explaining to the potential participants that their participation would be meaningful regardless of the parental or child problems felt by their families.

The third limitation is related to the exclusive use of self-answered questionnaires, not allowing us to access more detailed information, such as that obtained by interviews, or more reliable information, such as that obtained by the use of multiple sources and methods of data collection. This limitation also led to the fourth related limitation: the inexistence of a measure of social desirability, which makes it impossible to determine whether the results obtained reflect the reality or a more positive image of the participants. This effect may have been amplified by the fact that Portuguese governmental adoption agencies handled the invitation for participation in this study. However, to avoid this, the anonymity and confidentiality of the responses were ensured by an explicit indication that the adoption teams would not have access to the answers given in the questionnaires, nor would they be paired in any way with the identity of the participants or with documentary records of the adoption process.

The fifth limitation is related to the fact that our sample was composed of independent observations, mainly of female participants. Although our sample was composed of more men than usual in studies of the adoptive parent population (e.g., [41]), the supremacy of female participants and the independent nature of female and male participation may have biased the study results, especially regarding the moderating effect of gender. Future dyadic studies may be relevant to analyze the effect of gender on the proposed associations between these types of variables.

The sixth limitation is related to the collection of the study sample, which occurred during the COVID-19 pandemic. This context may have led to a bias in the results since it was a period that tended to evoke greater vulnerability and stress, especially concerning parenting [63]. However, we tried to overcome this limitation by collecting data related to the experience and impact of the pandemic on both parents’ and children’s lives.

A seventh limitation is linked to the high number of explanatory factors and interaction terms used in the regression models vs. the sample size, which may have reduced the statistical power of the analyses performed. Although the sample size of this study is adequate, this is a limitation that should be considered. In the future, it may be pertinent to replicate these analyses in even larger samples. We tried to overcome this limitation by using a less conservative significance level so as to not exclude a priori potential explicative factors for parenting stress in our results, which can be explored more appropriately in future studies. Finally, as an eighth and related limitation, it is important to mention the results concerning the marginal significance of self-compassion in explaining parenting stress. It would be important to investigate these data in further studies, possibly using a larger sample, while also investigating not only the independent contribution of the psychological resources under study but also their interdependence and their interaction with psychological variables other than those addressed in this study.

Regardless of the described limitations, this study presents several methodological and conceptual assets. Primarily, the involvement of all the Portuguese governmental adoption agencies in disseminating this study among adoptive parents made it possible to reach a high number of participants in our study. This translated into large representativeness in terms of different family dynamics (e.g., parents of only adopted children vs. parents of adoptive and biological children; families with a different number of adopted children; dual-parent vs. single-parent families), children’s developmental stages (e.g., parents of preschool vs. school-age children; parents of infants vs. children vs. adolescents), and family life cycle stages (e.g., first years post-adoption vs. adoptive placement several years ago), which allowed a comprehensive study of the relationship between the variables in question. Additionally, and as previously mentioned, the sample in this study included more men than is usual in parenting studies (e.g., [66]), particularly among adoptive parents (e.g., [41]), allowing for the analysis of the role played by the gender of the parents as a potential moderator of the relationship between the variables studied. In addition to this potential moderator, the heterogeneity of the sample also made it possible to study the potential moderating effect of the child’s age and the years passed since the adoptive placement, which is another innovation and added value of this study.

It is also necessary to highlight that this is the first study with adoptive parents to consider the role of these three psychological resources as potentially modifiable factors through psychological intervention and consequently focus on this research question. Although other studies have focused on understanding the impact of parenting-related characteristics on levels of parenting stress, they usually focus on sociodemographic characteristics, which cannot be modified through therapeutic intervention. Thus, in addition to being the first to focus on this research question, this study is also the first to understand the role played by characteristics of adoptive parents that can be modified in therapy for parenting stress.

### 4.5. Implications for Research and Clinical Practice

The knowledge gained in this study translates into several contributions to research and clinical practice with adoptive parents. Globally, the relationship established between parents’ mindfulness, psychological flexibility, self-compassion and parenting stress demonstrates the importance of including the promotion of these psychological resources in clinical intervention with adoptive parents, an area of intervention that has been increasingly recommended and is under development in several countries, and taking parents’ psychological resources into account in adoption research, a topic that has been poorly studied.

Regarding clinical practice with adoptive parents, the findings of our study are primarily important for remedial clinical intervention (i.e., with adoptive parents who are already dealing with higher levels of parenting stress) as they highlight the importance of including these three psychological resources as therapeutic goals with this population since these are resources on which clinical intervention can effectively produce effects [24] and that seem to contribute to a better adjustment of these parents. Considering our results about the potential moderators of the association between these psychological resources and parenting stress, the therapeutic work focused on parents’ mindfulness, self-compassion, and psychological flexibility seems to be capable of producing effects in reducing parenting stress regardless of the child’s age, the time passed since the adoptive placement or the parents’ gender, thus evidencing the transversality that this work can assume in this population. However, future research should explore whether decreases in parenting stress are effectively achieved by intervening in the promotion of these psychological resources and the moderators of the therapeutic response to treatment.

The role played by parents’ mindfulness, psychological flexibility, and self-compassion on parenting stress in our study also suggests the importance of assessing these psychological resources in individuals who are still candidates for or are in the course of the adoption process. This assessment may allow for the identification of individuals who are more likely to struggle in the future (i.e., individuals who show less evidence of possessing these resources), not to exclude them from the adoption process since these are psychological resources that can be easily improved through psychological intervention [67], but to include them in preventive interventions focused on cultivating these resources earlier in the adoption process to avoid high levels of parenting stress in the future.

Mindfulness- and self-compassion-based interventions are good examples of interventions aimed at promoting these psychological resources. If adapted for adoptive parents’ preferences and needs (e.g., targeting parenting stress, addressing real challenges faced by these parents), these interventions may be promising in equipping parents with techniques related to being in the present moment in the interactions with their children (fully listening and watching them), accepting aversive emotional states as part of the human condition (allowing the parents to maintain engaged in value-based behaviors even in the most challenging moments with their children), managing expectations about themselves as parents and the children they have vs. what they idealized, and being compassionate with oneself when they can’t act the way they would like to or when things don’t go the way they would like them to, as well as with the child in challenging moments (which, in turn, will contribute to a decrease in the self-criticism [another important intervention target with this parents]). These techniques can be worked out through the approach in therapy of content about the automatic pilot (parenting), doing versus being mode, seeing the child with a beginner’s mind, awareness of body sensations, watching the body during parenting stress, using self-compassion during stressful events, awareness and acceptance of parenting stress and how to respond rather than reacting to it. Although, future research testing the efficacy of such interventions is needed.

Regarding the implications for research, our results emphasize the relevance of parents’ psychological resources on adoptive parents’ adjustment and the pertinence of further studying the influence of mindfulness, self-compassion, psychological flexibility, and other related psychological resources (e.g., self-efficacy, resilience) on adoptive parents’ stress but also on other related outcomes (e.g., psychological adjustment, positive mental health).

Although many studies are still needed to fill several gaps in this field of research, the findings of this study provide preliminary evidence for the benefits of parents’ mindfulness, psychological flexibility, and self-compassion as resources potentially capable of preventing or reducing parenting stress and consequently promoting adoptive parents’ well-being. The mechanisms through which these psychological resources may act remain unknown and need to be further explored in the future.

## 5. Conclusions

Our findings provide relevant information for the promotion of adoptive families’ adjustment through psychological intervention and lay the groundwork for future research to replicate and extend our results. Therefore, the results of this study suggest that research, as well as clinical practice with adoptive parents in the pre- or postadoption period, should be devoted not only to the characteristics linked to the children, their history, the adoption process, and the sociodemographic and clinical characteristics of the parents but also to the characteristics that therapeutic intervention is capable of modifying, such as the psychological resources addressed in this study. Identifying and reducing the levels of parenting stress among adoptive parents is of the utmost importance, as it may lead to less emotional reactivity and, consequently, to more positive parenting practices [26], contributing to greater family harmony and healthier development of children. In turn, the innovative results of this study elucidate the importance that mindfulness, self-compassion, and psychological flexibility may have in reducing parenting stress and demonstrate the usefulness of cultivating them with (prospective and current) adoptive parents, particularly through their inclusion as target goals of psychological intervention both preventive and remedial.

## Figures and Tables

**Table 1:** Sociodemographic, Health-, Child-, and Adoption-Related Characteristics of the Sample: Descriptive Statistics.

Study Variables	Total Sample *N* = 302 N (%)
Parents-related	
Age (years); Mean (SD; Range)	46.62 (5.21; 34–66)
Sex	
Male	74 (24.5)
Female	228 (75.5)
Educational level	
Elementary/High school	90 (29.8)
University/Postgraduate degree	212 (70.2)
Professional status	
Employed	284 (94.0)
Unemployed or other	18 (6.0)
Marital status	
Single/Widower	57 (18.9)
Separated/Divorced	34 (11.3)
Married/Cohabitating	211 (69.9)
Type of relationship	
Opposite sex	211 (100)
Same sex	0 (0.0)
Number of household members; Mean (SD; Range)	3.26 (0.98; 1–8)
Household income	
Under EUR 3500/per month	244 (80.8)
Above EUR 3500/per month	58 (19.2)
Type of family	
Only adopted children	255 (84.4)
Both adopted and biological children	47 (15.6)
Diagnostic of mental health problems	
Never had	243 (80.5)
Had or currently has	59 (19.5)
Diagnostic of physical health problems	
Never had	229 (75.8)
Had or currently has	73 (24.2)
Infected with COVID-19	
Never	295 (97.7)
Currently or in the past	7 (2.3)
Risk population for COVID-19	
No	268 (88.7)
Yes	34 (11.3)
Child-related	
Age (years); Mean (SD; Range)	9.96 (3.79; 1–17)
Sex	
Male	163 (54.0)
Female	139 (46.0)
Health problems	
Physical or mental disability: yes	6 (2.0)
Physical health problems: yes	18 (6.0)
Special educational needs: yes	55 (18.2)
Mild/moderate behavior problems: yes	106 (35.1)
Severe behavior problems: yes	7 (2.3)
Diagnosed psychological/psychiatric problem: yes	23 (7.6)
Parents’ perceived impact of COVID-19 on child’s life; Mean (SD; Range) ^1^	21.85 (3.71; 8–34)
Adoption-related	
Application type	
Single	67 (22.2)
Couple	235 (77.8)
Number of children adopted at the same time; Mean (SD; Range)	1.22 (0.49; 0–4)
Child’s age at adoptive placement (years); Mean (SD; Range)	4.27 (3.05; 0.21–15.42)
Time since child’s adoptive placement (years); Mean (SD; Range)	6.22 (3.51; 0.1–17.2)
Years in foster care; Mean (SD; Range)	2.40 (1.79; 0–11)
Reason for foster care measure	
Child maltreatment	159 (60.0)
Other adverse life experiences	106 (40.0)
Residential hosting	
No/Don’t know	11 (3.8)
Yes	278 (96.2)

^1^ The perceived impact of COVID-19 was measured through a bipolar adjective scale (ranging from 0—very negative impact to 4—very positive impact) in which higher and lower values indicated, respectively, a greater positive and a greater negative perceived impact of COVID-19 in the participants’ lives.

**Table 2:** Descriptives Statistics and Pearson’s Correlations for the Variables under Study.

	Descriptives	Correlations			
**Variables**	*Mean (SD; Range)*	1	2	3	4
Parenting Stress	69.40 (21.65; 37–148)	-			
2. Mindfulness	72.36 (13.61; 15–90)	−0.47 ***	-		
3. Self-compassion	3.64 (0.70; 1.29–5)	−0.51 ***	0.58 ***	-	
4. Psychological inflexibility	16.06 (7.88; 7–47)	0.53 ***	−0.50 ***	−0.73 ***	-

Note. *** *p* < 0.001.

**Table 3:** Hierarchical Regression Models Explaining Parenting Stress.

	Exploratory Model ^1^				Final Model ^2^
Variables	Step 1: Parent-Related, Δ *R*^2^ *=* 0.10 *R*^2^_adj_ = 0.08	Step 2: Child-Related, Δ *R*^2^ *=* 0.19 *R*^2^_adj_ = 0.27	Step 3: Psychological Resources, Δ *R*^2^ = 0.21 *R*^2^_adj_ = 0.47	Step 4: Hypothesized Moderators, Δ *R*^2^ = 0.01 *R*^2^_adj_ = 0.48	*R*^2^_adj_ = 0.49
	*beta* (SE)	*beta* (SE)	*beta* (SE)	*beta* (SE)	*beta* (SE)
Parents’ educational level	6.05 (0.13) *	4.52 (0.10) ^$^	2.44 (0.05)	2.76 (0.06)	
Parents’ diagnosis of PHP ^3^	2.85 (0.06)	−0.58 (−0.01)	1.69 (0.03)	1.65 (0.03)	
Parents’ diagnosis of MHP ^4^	7.95 (0.15) *	8.05 (0.15) **	−2.61 (−0.05)	−2.75 (−0.05)	
Perceived impact of COVID-19 on parents’ life	−1.73 (−0.23) ***	−0.50 (−0.07)	−0.19 (−0.02)	−0.17 (−0.02)	
Child’s severe behav. probl.		41.56 (0.29) ***	30.44 (−21) ***	29.77 (0.21) ***	31.11 (0.21) ***
Child’s diagnosis of psychol./psychi. probl.		13.40 (0.16) **	14.62 (0.18) ***	13.24 (0.16) **	12.96 (0.16) **
Perceived impact of COVID-19 on child’s life		−1.31 (−0.22) ***	-0.81 (−0.14) *	−0.79 (−0.13) *	−0.94 (−0.16) ***
Parents’ mindfulness			−0.33 (−0.21) ***	−0.32 (−0.20) ***	−0.34 (−0.21) ***
Parents’ psychol. flexibility			0.74 (0.27) ***	0.76 (0.28) ***	0.70 (0.25) ***
Parents’ self-compassion			−3.94 (−0.13) ^$^	−3.73 (−0.12) ^$^	−3.87 (−0.13) ^$^
Parents’ gender				1.18 (0.02)	
Child’s age (years)				0.89 (0.16) **	0.55 (0.10) *
Time since child’s adoptive placement (years)				−0.49 (−0.08)	
Constant	94.83 ***	100.85 ***	113.20 ***	104.26 ***	110.00 ***

Note. ^$^ *p* < 0.10; * *p* < 0.05; ** *p* < 0.01; *** *p* < 0.001. ^1^ Hierarchical Regression Model; all study variables; enter method; all steps visible in the table. ^2^ Hierarchical Regression Model; only with the significant variables in the exploratory model; enter method; only the final step is visible in the table. ^3^ Physical health problems. ^4^ Mental health problems.

## Data Availability

The data presented in this study are available on request from the corresponding author. The data are not publicly available due to ethical reasons.
